# Imbalanced Expression of Th2 and Treg Cell-related Parameters in Peripheral Blood Mononuclear Cells in Patients with Allergic Asthma

**Published:** 2018-01

**Authors:** Samane Hoseini-Shahrestanak, Nasrin Bazargan, Leila Rahimian, Maryam Nemati, Saeed Solaymani, Abdollah Jafarzadeh

**Affiliations:** 1 Department of Immunology, Medical School, Kerman University of Medical Sciences, Kerman, Iran.,; 2 Department of Internal Medicine, Medical School, Kerman University of Medical Sciences, Kerman, Iran.,; 3 Department of Laboratory Sciences, Para-Medical School, Kerman University of Medical Sciences, Kerman, Iran.,; 4 Department of Immunology, Medical School, Rafsanjan University of Medical Sciences, Rafsanjan, Iran.

**Keywords:** Allergic Asthma, Th2, Treg, Transcription Factors, GATA3, FOXP3

## Abstract

**Background:**

The imbalance between Th2 and Treg cells plays fundamental role in the pathogenesis of allergic asthma. The current study aimed at assessing the expression of some Th2 and Treg cell-related parameters in patients with allergic asthma.

**Material and Methods:**

The serum and peripheral blood mononuclear cell (PBMC) samples were collected from 30 patients with asthma and 36 healthy subjects. The serum levels of transforming growth factor (TGF)-β, interleukin (IL)-4, as well as the expression levels of *GATA3* and *FOXP3* genes in PBMCs were determined by the enzyme-linked immunosorbent assay (ELISA) and real-time polymerase chain reaction (PCR), respectively. The PBMCs were cultured for 48 hours with/without phytohemagglutinin (PHA) stimulation. The TGF-β and IL-4 levels in supernatants were also determined.

**Results:**

The serum levels of IL-4, the expression level of *GATA3*, and *GATA3/FOXP3* ratio in patients with asthma were significantly higher than healthy subjects (P <0.002, P <0.001, and P <0.004, respectively). The *FOXP3* expression did no differ between the two groups. The serum level of TGF-β as well as its secretion profile in non-stimulated and stimulated PBMCs isolated from patients with asthma were significantly higher than those of the controls (P <0.03, P <0.001, and P <0.001, respectively). The serum TGF-β levels in severe asthma were significantly higher than moderate asthma; whereas the TGF-β secretion by PHA-stimulated PBMCs isolated from moderate asthma was higher than that of severe pattern of the disease (P <0.001 and P <0.05, respectively). The *GTAT3/FOXP3* expression ratio in moderate asthma was significantly higher than severe form (P <0.04).

**Conclusion:**

The results confirmed a Th2 cell-biased pattern and possible contribution of TGF-β in allergic asthma. TGF-β may have different expression patterns in moderate and severe asthma and the two forms of the disease may have differences in some main immunological parameters.

## INTRODUCTION

The asthma is the most prevalent chronic lung disease characterized by bronchial hyper-responsiveness, airway inflammation, and reversible airflow obstruction, and its clinical symptoms vary from mild to severe (1, 2). Approximately 300 million individuals have asthma worldwide, and about 250,000 deaths are attributed to the disease each year (1, 3). Different cellular elements from both innate/natural and adaptive immune systems participate in the pathogenesis of allergic asthma, particularly such as mast cells, eosinophils, lymphocytes (T- and B-cells), macrophages, and neutrophils (4). The allergic asthma can be classified into several subgroups, including clinical, pathological, and molecular patterns. The Th2 cell-dependent asthma is one of the considerable molecular diagrams of allergic asthma (5). The Th2 lymphocytes play fundamental role in the pathogenesis of the allergic asthma via the releasing of cytokines, particularly interleukin (IL)-4, IL-5, and IL-13 (6, 7). IL-4 leads to the IgE formation by B-cells and IgE-mediated mast cell activation plays a pivotal role in the pathogenies of allergic asthma (8). IL-5 causes eosinophils aggregation into the site of inflammation (9). IL-13 is a multifunctional cytokine that raises airway hyper-responsiveness, airway mucus hyper secretion, goblet cell hyperplasia, and fibrosis (8).

The binding of IL-4 to its receptor results in the phosphorylation of signal transducer and activator of transcription 6 (STAT-6), and ultimately induces the expression of Th2 related-master transcriptional factor *GATA3* (10). Human *GATA3* gene, located on chromosome 10, is an important element in Th2 cell development and acts as an inducer to synthesize Th2-related cytokines (11). GATA3 was verified as a Th2 -related master transcriptional factor that induces the production IL-4, IL-5, and IL-13 in Th2 cells (12).

The Treg cells constitute 5% to 15% of the total circulating CD4^+^ T lymphocytes divided into two subsets. The natural Tregs (nTreg) cells are generated in the thymus, whereas the induced Treg (iTreg) cells are differentiated from peripheral CD4^+^ T lymphocytes after antigenic recognition and the existence of IL-2 and TGF-β (13, 14). The immunosuppressive effects of Treg cells are exerted by releasing TGF-β, IL-10, and IL-35 that are necessary to establish tolerance to self-constituents or foreign antigens (13, 15). The FOXP3 protein is considered as a master transcriptional factor of Treg cells and its gene is mapped to the X chromosome (15, 16). Genetic abnormalities in *FOXP3* gene result in the inflammatory syndrome named immune dysregulation, polyendocrinopathy, enteropathy, and X-linked (IPEX) (17).

There is evidence indicating that Th2/Treg imbalance plays an essential role in the development of allergic sicknesses (18). The results of a number studies indicated that the count of Th2 cells was enhanced, while the count of Treg cells was diminished in the peripheral blood of patients with asthma or in the models of experimentally-induced allergic asthma, representing the contribution of the Th2/Treg imbalance in the pathogenesis of allergic asthma (7, 19).

It should be noted that the measurement of transcription factors GATA3 and FOXP3 may more reliably indicate the Th2/Treg cell development than the single assessment of a Th2 or Treg cell-related element. Therefore, it seems that the measurement of the transcriptional factor level is more important to evaluate the balance in Th2/Treg immune responses.

There are some controversies regarding the association of Th2 and Treg cell-related parameters with asthma severity. For example, the results of a number of studies demonstrated decreased serum level of TGF-β in patients with asthma (19, 20), while others reported elevated levels of this cytokine in the patients (21–23). Moreover, the exact association of Th2 and Treg cell-related parameters with asthma severity is still unclear.

The current study aimed at determining the key parameters of Th2 (IL-4 and GATA3) and Treg (TGF-β and FOXP3) cells in the peripheral blood of patients with asthma and evaluating the production of Th2- and Treg-type cytokines (IL-4 and TGF-β) by unstimulated and stimulated peripheral blood mononuclear cells (PBMCs) isolated from patients after in vitro culturing. Moreover, the association of the aforementioned parameters between moderate and severe asthma was evaluated to clarify the possible relationship and provide new insights into the asthma immunopathogenesis.

## MATERIALS AND METHODS

### Subjects

From September 2015 to September 2016, blood specimens were collected from 30 patients with allergic asthma referred to Allergy Unit of Afzalipour Hospital affiliated to the Kerman University of Medical sciences, Kerman, Iran. The patients were visited by expert allergists, their asthma was verified by the specialist, and the diagnosis was confirmed according to the standard criteria. The severity of asthma was also determined based on the global initiative for asthma (GINA) criteria as moderate or severe patterns (24). None of the patients received systemic glucocorticoids at least one month prior to investigation and no subject treated with other immunosuppressant agents. The subjects with asthma had no history of smoking.

Totally, 36 healthy nonsmoker subjects were enrolled in the study as the control group. The healthy individuals were recruited among blood donators of the Blood Transfusion Organization of Kerman and none of them had an acute or chronic sickness. The control subjects were in good general health status, without history of respiratory disorders or other relevant diseases. Indeed, each person with cigarette smoking, medication, and disorders (such as the history of recurrent infections, malignancy, and any suspicious immunological diseases) was excluded from the study. Other exclusion criteria were the history of surgery and major trauma within the six months prior to blood collection. The Ethics Committee of Kerman University of Medical Sciences confirmed the study protocol, and the informed written consent was obtained from the participants before enrollment.

### Preparation and culturing of PBMCs

PBMCs were isolated from fresh heparinized venous blood using density-gradient centrifugation technique (Lymphosep, Biosera, UK). Then, the isolated PBMCs were washed and re-suspended in RPMI (Roswell Park Memorial Institute) 1640 medium (Biosera, UK). The PBMCs obtained from each person were divided into two parts as follows: one part was used to culture the cells, and measure the production of TGF-β and IL-4 by unstimulated and stimulated PBMCs; the other part was used to extract RNA and trace *GATA3* and *FOXP3* expression.

### Culturing PBMCs

About 1×10^6^ PBMCs from healthy volunteers and patients with asthma were cultured in 24-well plates in RPMI 1640 supplemented with 10% heat-inactivated fetal bovine serum (Gibco Life Technologies, Paisley, UK), 100 U/mL penicillin, 100 μg/mL streptomycin, and 2 mM/L L-glutamine. The PBMCs were cultured with/without phytohemagglutinin (PHA, 10 μg/mL; Gibco) stumulation for 48 hours at 37°C in 5% CO_2_ and then, the supernatants of PBMCs were collected and stored at −40°C to measure TGF-β and IL-4.

### RNA extraction and real-time polymerase chain reaction (PCR)

Total RNA was isolated from PBMCs using Trizol reagent (Bionner, Korea) according to the manufacturer’s instructions. The extracted RNA quality was assessed by running on the agarose gel, pre-treated with ethidium bromide, using electrophoresis. The RNA purity was assessed by measuring its absorption at 260 and 280 nm, using a spectrophotometer system (Nano-Drop, Wilmington, USA).

The complementary DNA (cDNA) synthesis kits (Bionner, Korea) were used to convert the target RNA into the cDNA. The reverse transcription amplification protocol was as follows: 70°C for 10 minutes (in the absence of reverse transcriptase enzyme), 20°C for 1 minute (cooling phase), addition of reverse transcriptase enzyme at 42°C for 60 minutes, and eventually the protocol was finished by a step at 95°C for 10 minutes to inactivate the reverse transcriptase enzyme.

Real-time PCR protocol was conducted to estimate *GATA3* and *FOXP3* genes expression by utilizing a 7300 Real–Time PCR System (Applied Biosystems, USA). The real-time PCR reaction was prepared by adding SYBR Green PCR Master Mix (Bionner, Korea), 200 ng of template cDNA, and 2 μL of gene specific primers (Table 1) from working stocks (10 pmol/μL). The thermal cycling program was entailed: an initial heating at 95°C for 15 minutes, 40 cycles at 95°C for 30 seconds, 72°C for 40 seconds, and eventually 72°C for 60 seconds.

**Table 1.** The used primers for the gene expression of T-bet, GATA3 and FOXP3 by PBMCs from healthy subjects and asthmatic patients.

**Gene**	**Primer**
GATA-3	Forward: 5-AGCCAGGAGAGCAGGGACG-3 Reverse: 5-CTGTTAATATTGTGAAGCTTGTAGTAGAG-3
FOXP3	Forward: 5-GAACGCCATCCGCCACAACCTGA-3 Reverse: 5-CCCTGCCCCCACCACCTCTGC-3
β-Actin	Forward: 5-GCCGGGACCTGACTGACTAC-3 Reverse: 5-TTCTCCTTAATGTCACGCACGAT-3

To normalize the amplified cytokine target genes, the β-actin gene was used as housekeeping gene. The amount of *GATA3* and *FOXP3* expression in the isolated PBMCs was assessed as units relative to the amount of β-actin expression calculated by the 2^−ΔΔ^_Ct_ formula. The PCR products were electrophoresed on 1% agarose gel after staining with 0.5 mg/mL ethidium bromide.

### The serum and PBMC supernatant levels of TGF-β and IL-4

The serum and PBMC supernatant levels of TGF-β and IL-4 were quantified using commercial ELISA (the enzyme-linked immunosorbent assay) kits for human TGF-β and IL-4 (eBioscience, USA).

### Serum IgE concentrations

Serum total IgE concentrations (IU/mL) were measured by the commercial ELISA kit (Monobind Inc., Costa Mesa, CA, USA).

### Statistical analysis

Data were expressed as mean ± standard error of the mean (SEM). Differences in variables were analyzed by appropriate statistical tests including ANOVA, the Student *t*, Kruskal-Wallis, and Mann-Whitney U tests; P values <0.05 were regarded statistically significant.

## RESULTS

The mean age of the patients with asthma and the healthy subjects were 36.7 ± 14.08 and 39.88 ± 11.83 years, respectively. There was no statistically significant difference in terms of age between the patients with asthma and healthy individuals (P=0.32). The gender distribution of patients was 16 (53.3%) females and 14 (46.7%) males, and in the control group it was 20 (55.6%) females and 16 (44.4%) males (P=0.52).

### Serum IgE concentration in patients with asthma and healthy individuals

The serum total IgE concentrations in patients with asthma were significantly higher than those of the healthy controls (289.77 ± 228.01 IU/mL vs. 54.01 ± 27.09 IU/mL, P <0.001). The total IgE concentration in both groups of patients with severe asthma (364.30 ± 273.54 IU/mL) and moderate asthma (246.63 ± 191.91 IU/mL) were significantly higher than that of the healthy controls (P <0.001). There was no significant difference in terms of serum IgE level between patients with severe and moderate asthma, although the immunoglobulin level was higher in patients with severe asthma (P=0.17).

### Serum levels of TGF-β and IL-4 in patients with asthma and healthy controls

**Figure 1.**
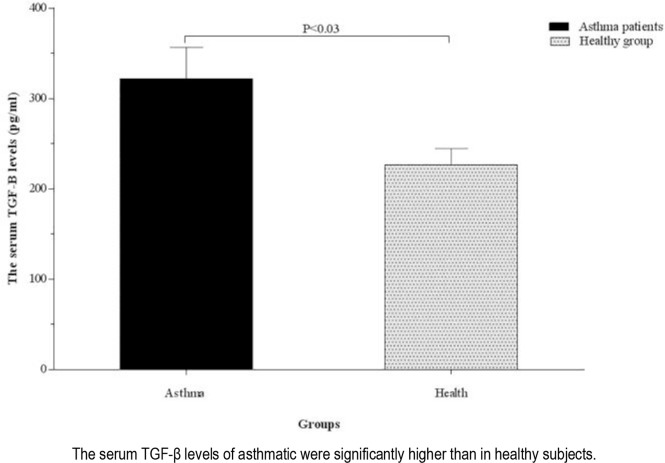
Comparison of the serum TGF-β levels between healthy group and asthmatic patients.

**Figure 2.**
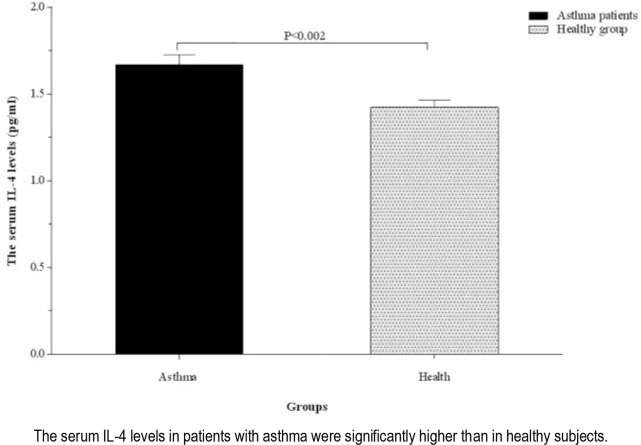
Comparison of the serum IL-4 levels between healthy group and asthmatic patients.

**Figure 3.**
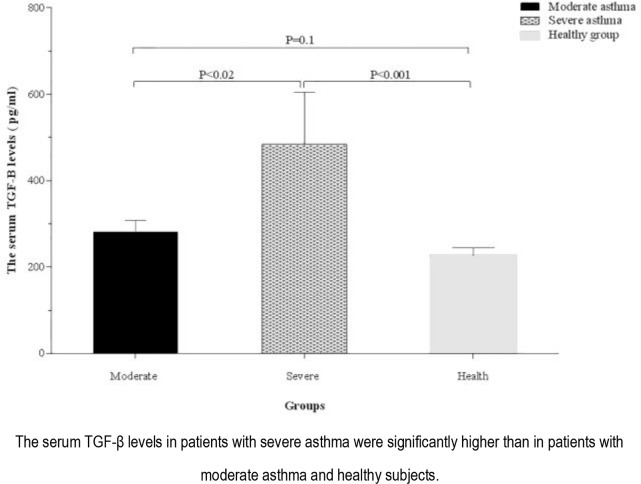
Comparison of the serum TGF-β levels between healthy group and asthmatic patients according to disease severity

The serum levels of TGF-β and IL-4 were significantly higher in patients with asthma than the healthy controls (321.70 ± 35.12 vs. 226.69 ± 17.81 pg/mL, P <0.03 and 1.66 ± 0.05 vs 1.42 ± 0.04 pg/mL, P <0.002; respectively) (Figures 1 and 2). The serum levels of TGF-β were higher in patients with severe asthma (484.25±120.73 pg/mL) than the ones with moderate asthma (281.07±27.31 pg/mL, P <0.02) and healthy controls (226.69±17.81 pg/mL, P <0.001). There was no significant difference in terms of the serum level of TGF-β between patients with moderate asthma and healthy controls, although the parameter was higher in patients with moderate asthma (P=0.10) (Figure 3).

**Figure 4.**
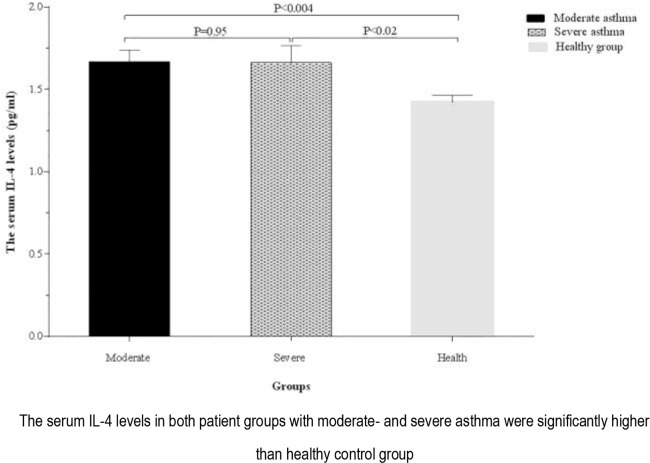
Comparison of the serum IL-4 levels between healthy group and asthmatic patients according to disease severity.

The serum levels of IL-4 in patients with moderate (1.66±0.06 pg/mL) and severe asthma (1.66 ± 0.10 pg/mL) were significantly higher than that of the healthy controls (1.42 ± 0.04 pg/mL; P <0.004 and P <0.02, respectively). No significant difference was observed in terms of the serum level of IL-4 between patients with moderate and severe asthma (P=0.95) (Figure 4).

### The FOXP3 and GATA3 expression and their ratio in PBMCs isolated from patients with asthma and healthy individuals

The *GATA3* expression in PBMCs isolated from patients with asthma was significantly higher than that of the healthy subjects (3.30±0.51 vs. 1.15±0.10, P<0.001). The *FOXP3* expression in PBMCs isolated from patients with asthma was also higher than that of the healthy subjects, but the difference was not significant (2.15±0.76 vs. 1.01±0.11, P=0.10). *GATA3/FOXP3* ratio in PBMCs isolated from patients with asthma was significantly higher than that of healthy subjects (9.49±2.02 vs. 3.48±0.78, P<0.004).

**Figure 5.**
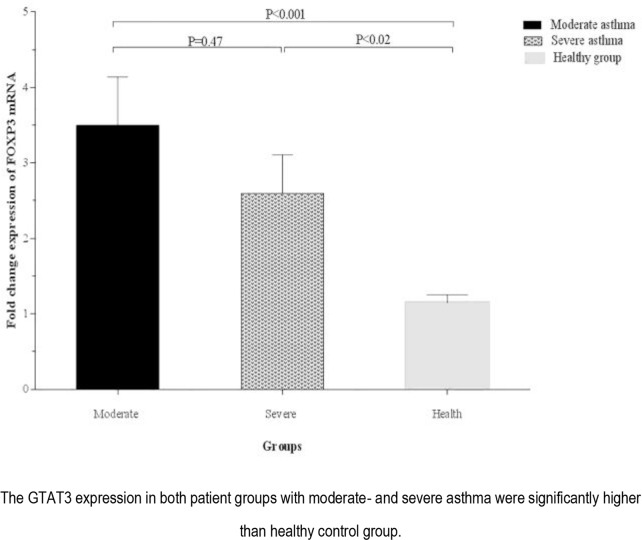
Comparison of the expression of GATA3 by isolated PBMCs from healthy group and asthmatic patients according to disease severity.

The expression of *GTAT3* in PBMCs isolated from patients with moderate (3.50±0.64) and severe asthma (2.60±0.51) were significantly higher than that of the healthy controls (P<0.001 and P<0.02, respectively). There was no significant difference in terms of *GATA3* expression between patients with moderate and severe asthma (Figure 5).

**Figure 6.**
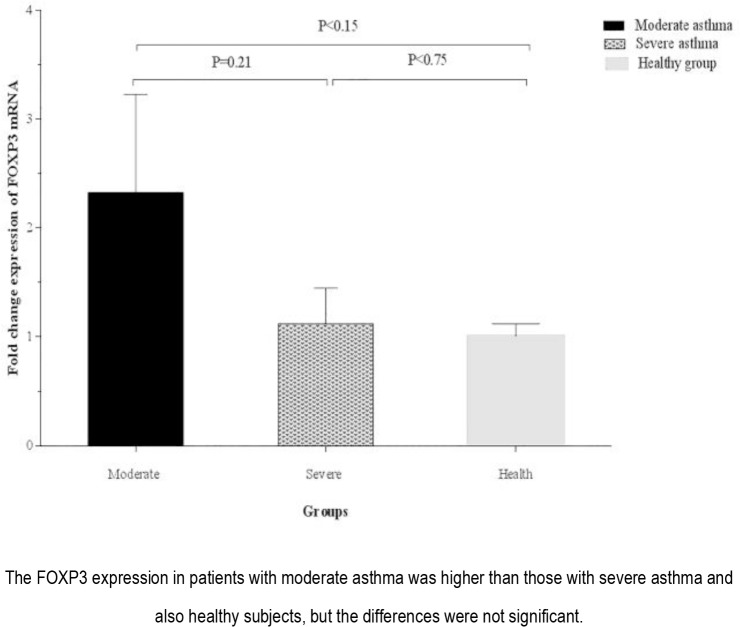
Comparison of the expression of FOXP3 by isolated PBMCs from healthy group and asthmatic patients according to disease severity.

The expression of *FOXP3* in PBMCs isolated from patients with moderate asthma (2.33±0.90) was higher than that of the ones with severe asthma (1.12±0.33) as well as the healthy subjects, but the differences were not statistically significant (Figure 6).

**Figure 7.**
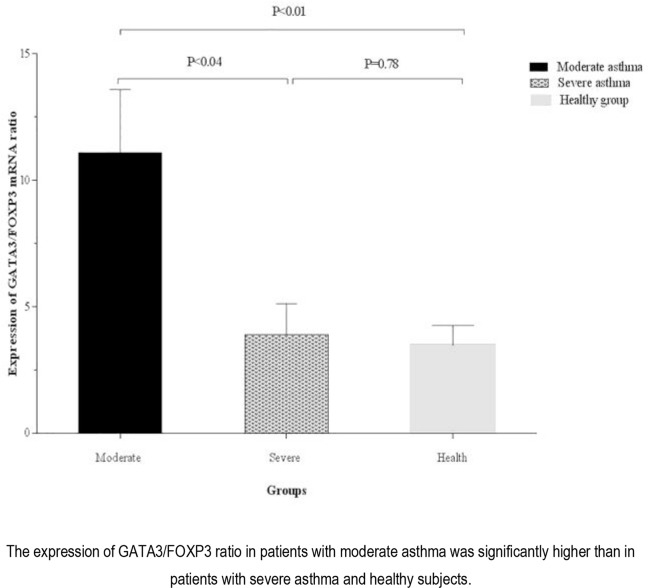
Comparison of the expression of GATA3/FOXP3 ratio by isolated PBMCs from healthy group and asthmatic patients according to disease severity.

The *GTAT3/FOXP3* expression ratio in PBMCs isolated from patients with moderate asthma (11.09 ± 2.50) was significantly higher than that of the patients with severe asthma (3.90 ± 1.22) and the healthy controls (3.48 ± 0.78), (P<0.04 and P <0.01, respectively). There was no significant difference in terms of GTAT3/FOXP3 expression ratio between patients with severe asthma and healthy subjects (Figure 7).

### TGF-β and IL-4 production by unstimulated and PHA-stimulated PBMCs in patients with asthma and healthy subjects

**Figure 8.**
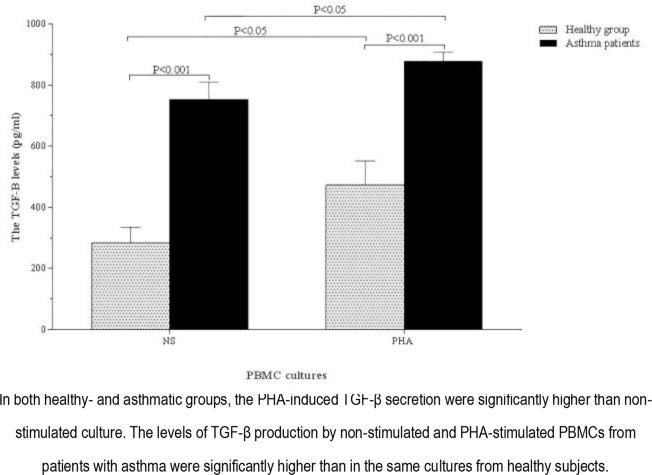
TGF-β production by non-stimulated and PHA-stimulated PBMCs from the healthy group and asthmatic patients.

**Figure 9.**
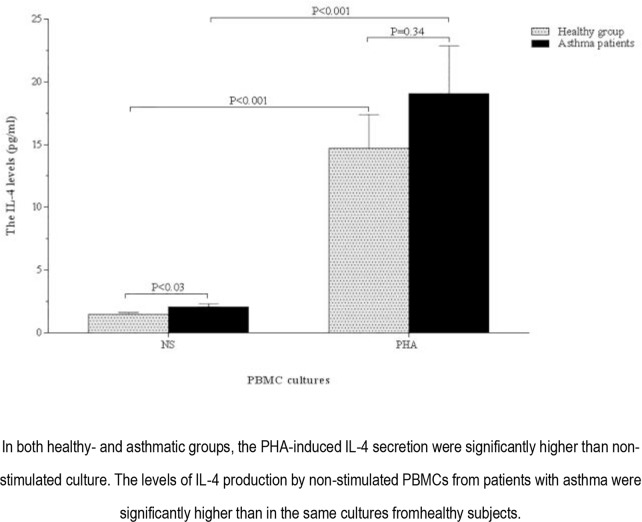
IL-4 production by non-stimulated and PHA-stimulated PBMCs from the healthy group and asthmatic patients.

The amounts of TGF-β and IL-4 production by unstimulated and PHA-stimulated PBMCs in patients with asthma and healthy individuals are shown in figures 8 and 9 as well as Table 2.

**Table 2.** The secretion levels of TGF-β and IL-4 by non-stimulated and PHA-stimulated PBMCs from asthmatic patients and healthy group according to disease severity.

**Groups**	**Stimulator of PBMC**	**Grade**	**Number**	**TGF-β levels (Pg/ml)**	**IL-4 levels (Pg/ml)**
**Asthmatic patients**	Without stimulation	Moderate	19	775.51 ± 68.45	2.16 ± 0.33
		Severe	11	718.27±110.57	1.94 ± 0.37
		Total	30	752.61 ± 57.26	2.07 ± 0.23
	PHA	Moderate	19	858.42 ± 42.66	19.66 ± 4.51
		Severe	11	660.47±102.53	16.33 ± 7.62
		Total	30	805.63 ± 45.84	19.05 ± 3.81
**Healthy group**	Without stimulation	------	36	283.75 ± 51.42	1.48 ± 0.15
	PHA	------	36	418.56 ± 77.72	14.68 ± 2.7

In both groups of healthy subjects and patients with asthma, TGF-β and IL-4 production by PHA-stimulated PBMCs were significantly higher than those of the unstimulated cells (P <0.05 and P <0.001, respectively) (Figures 8 and 9).

The levels of TGF-β and IL-4 production by non-stimulated PBMCs in patients with asthma were significantly higher than those of healthy controls (P <0.001 and P <0.03, respectively) (figures 8 and 9). Moreover, the production of PHA-stimulated TGF-β by PBMCs isolated from patients with asthma was significantly higher than that of healthy controls (P <0.001) (Figure 8). There was no significant difference in terms of the production of PHA-stimulated IL-4 between patients and healthy individuals, although the parameter was higher in patients groups (P=0.34) (Figure 9).

The production of TGF-β by unstimulated and PHA-stimulated PBMCs isolated from patients with moderate asthma were higher than those of the ones with severe asthma, although the difference was significantly higher in PHA-stimulated cells (P <0.05) (Table 2). No significant difference was observed between patients with moderate and severe asthma regarding the production of IL-4 by non-stimulated and PHA-stimulated PBMCs (Table 2). There was no significant difference between males and females in patients or in the control groups regarding the production of TGF-β and IL-4 by unstimulated and PHA-stimulated PBMCs (Table 3).

**Table 3.** The secretion levels of TGF-β and IL-4 by non-stimulated and PHA-stimulated PBMCs from asthmatic patients and healthy group according to their gender.

**Groups**	**Stimulator of PBMC**	**Gender**	**Number**	**TGF-β levels (Pg/ml)**	**IL-4 levels (Pg/ml)**
**Asthmatic patients**	Without stimulation	Men	14	778.29±110.25	2.25 ± 0.46
		Women	16	735.49± 69.50	1.95 ± 0.28
		Total	30	752.61 ± 57.26	2.07 ± 0.23
	PHA	Men	14	821.56 ± 71.24	20.46 ± 5.57
		Women	16	791.69 ± 63.36	17.88 ± 5.64
		Total	30	805.63 ± 45.84	19.05 ± 3.81
**Healthy group**	Without stimulation	Men	16	298.11 ± 52.59	1.43 ± 0.16
		Women	20	274.17 ± 82.35	1.51 ± 0.22
		Total	36	283.75 ± 51.42	1.48 ± 0.15
	PHA	Men	16	443.51± 137.28	13.99 ± 3.33
		Women	20	396.73 ± 91.51	15.37 ± 4.51
		Total	36	418.56 ± 77.72	14.68 ± 2.7

## DISCUSSION

The results of the current study indicated that the serum level of IL-4 and *GATA3* expression were significantly higher in patients with asthma compared with the healthy individuals. The current study results confirmed a Th2 cell-biased pattern in patients with asthma that was consistent with the data obtained from other studies showing an excessive Th2 immune response in allergic asthma (25). The increased serum IL-4 levels and elevated expression of *GATA3* in patients with asthma confirmed a deviation toward Th2 cells in patients with asthma that play a prominent role in pathogenesis of allergic asthma.

The results of the current study also showed that the serum levels of TGF-β in patients with asthma were significantly higher than those of the healthy individuals. There were some controversies regarding the TGF-β levels in patients with asthma. The results of some studies demonstrated reduced serum levels of TGF-β in patients with asthma (19, 20), while others reported elevated levels of this cytokine in such patients (21–23). The reasons for these disagreements remain to be explained in further studies. These discrepancies may be due to the variations in some inclusion criteria such as asthma severity, age, gender, treatment program, race and ethnicity, or even geographical parameters. Interestingly, in a complementary set in the current study protocol, it was observed that in parallel to elevated serum levels of TGF-β in patients with asthma, the amounts of TGF-β production by unstimulated and PHA-stimulated PBMCs isolated from patients with asthma were significantly higher than those of the healthy controls. The current study results reciprocally confirmed each other.

A number of pro-inflammatory and anti-inflammatory properties were attributed to TGF-β (26). In the bronchial airways, TGF-β was produced by a number of residential and infiltrated cells such as epithelial, fibroblasts, endothelial cells, smooth muscle cells, eosinophils, macrophages, and lymphocytes (26). The aforementioned cells secrete TGF-β; it may be considered as a reason for the elevated serum levels of TGF-β in patients with asthma. The TGF-β may contribute to immunopathogenesis of allergic asthma through recruitment of leukocytes such as macrophages and granulocytes into bronchial airways (27, 28), induction of the polarization of naïve CD4^+^ T-cells into effector inflammatory Th17- and Th9-cells (28), triggering of remodeling processes, and induction of the expression of some matrix metalloproteinases (29). The beneficial effect of anti-TGF-β antibody is demonstrated in some murine models of asthma (30).

In the current study, *FOXP3* expression did not significantly differ between patients with asthma and healthy subjects. The *FOXP3* is expressed in Treg cells needed for the exertion of their immunosuppressive effects (31). The current study data regarding *FOXP3* expression by PBMCs indicated that there may be no functional impairment in Treg cells of patients with asthma. Treg cells may have normal activity in patients with asthma, but the effector T cells involving in the asthma pathogenesis (such as Th2 cells) may be non-responder to the suppressive effects of Treg cells. The normal or even elevated number of Treg cells with acceptable suppressive activity was reported in patients with atopic dermatitis or allergic rhinitis; diseases with similar immunopathogenesis to allergic asthma (32).

The results of the current study also indicated that *GATA3/FOXP3* expression ratio in patients with asthma was significantly higher than those of healthy individuals. Since GATA3 and FOXP3 are the principal transcription factors of Th2 and Treg cells, respectively, *GATA3/FOXP3* mRNA expression ratio may be considered as a useful substitute parameter to determine Th2/Treg cells status in patients with asthma. Therefore, the occurrence of an imbalance in the levels of Th2/Treg cell-related transcription factor with a deviation in the direction of Th2 cell may contribute to the development of the allergic asthma. It is necessary to maintain the balance between Th2 and Treg cell to prevent allergic disorders. It is indicated that occurrence of an imbalance between Th2/Treg cells results in the development of a number of disorders such as atopic and allergic diseases (19). Therefore, the correction of Th2/Treg imbalance using effective immunotherapeutic agents (probably at levels of cytokines, receptors, or signaling pathways) may be an interesting investigation field in future studies. It is indicated that the suppression of STAT6 using small interfering RNA (siRNA) improves the Th2/Treg ratio in patients with allergic rhinitis (33, 34). Similar immunotherapeutic programs such as GATA3 can introduce novel therapeutic agents to modulate inflammation in patients with asthma.

The current study results also demonstrated that in both patients with moderate and severe asthma, serum levels of IL-4 and *GTAT3* expression were higher than those of the healthy individuals. There were no significant differences in serum levels of IL-4 and *GATA3* expression between patients with moderate and severe asthma. These results indicated that the Th2 immune responses contribute to the pathogenesis of both moderate and severe patterns of allergic asthma.

The results of the current study also indicated that the serum levels of TGF-β in patients with severe asthma were higher than those of the ones with moderate asthma. However, in controversy to serum levels of TGF-β, the production of this cytokine by PHA-stimulated PBMCs isolated from patients with moderate asthma was higher than that of the ones with severe asthma. These differences may be attributed to different in vivo cell producers of TGF-β. As mentioned above, TGF-β is produced by some non-lymphoid cells (such as epithelial, fibroblasts, endothelial, and smooth muscle cells) and a number of leukocytes such as eosinophils, macrophages, and lymphocytes in vivo. The aforementioned cells may be responsible for increased serum levels of TGF-β in patients with severe asthma. TGF-β is mainly produced by Treg cells within the PBMC population in vitro. Increased production of TGF-β by PHA-stimulated PBMCs isolated from patients with moderate asthma may be attributed to higher frequency of Treg cells in subjects with moderate asthma in comparison with those with severe asthma. It should be also noted that TGF-β may perform different roles in moderate and severe forms of asthma, on the basis of its concentration. As mentioned above, some pro-inflammatory and anti-inflammatory effects are attributed to TGF-β production (26). In patients with moderate asthma, TGF-β may act as a modulator of inflammatory responses and prevent severe immunopathologic responses, due to overcoming its anti-inflammatory properties. In higher levels as observed in patients with severe asthma, TGF-β may accelerate immunopathologic responses, due to overcoming its pro-inflammatory properties. In the presence of IL-6, TGF-β, which induces the expression of *FOXP3,* is downregulated and *RORγt* (a principle transcription factor of Th17) expression is upregulated, which result in the differentiation of Th17 cells (35, 36). Th17 cells produce some pro-inflammatory cytokines, particularly IL-17A, IL-17F, IL-21, IL-22, TNF-α, and GM-CSF, which may have important roles in the reinforcement of severe form of asthma. Th17 cells may promote the development of severe allergic asthma due to their link to airway neutrophilia (37). Therefore, TGF-β may also influence the severity of allergic asthma. Accordingly, targeting TGF-β or its related signaling pathways may lessen asthma severity.

However, the current study results demonstrated that *GTAT3/FOXP3* expression ratio in patients with moderate asthma was significantly higher than that of the subjects with severe asthma. Therefore, it seems that moderate and severe asthma may have differences in some important immunological aspects. Other pathological mechanisms of Th2-dependent inflammation (such as Th17, Th9, and Th22, or even Th1 immune responses) may also contribute to the pathogenesis of severe asthma. Indeed, high levels of Th1 cytokines are found in humans and mice with severe asthma (38). Therefore, a wide range of immunopathological responses may involve in the development of severe asthma. Accordingly, a combination of therapies is needed to control asthma severity and prevent its exacerbations.

In the current study, one Th2 cell-associated cytokine (IL-4) was measured. Since IL-4 secretion is an indicator for Th2 immune responses, other Th2 cells-associated cytokines (including IL-5 and IL-13) may express same pattern as observed for IL-4. Moreover, the flow of cytometric analysis of T-cell subsets and evaluation of the other immunological parameters such as chemokines and toll-like receptors were not a part of the current study protocol, which should be investigated in future studies.

In conclusion, elevated serum levels of IL-4 and increased expression of *GATA3* and *GATA3/FOXP3* ratio in PBMCs isolated from patients with asthma indicate an imbalance in the levels of Th2/Treg-related transcription factors and a Th2-deviated pattern in patients with asthma. The improvement of Th2/Treg immune responses should be considered more to design effective therapeutic strategies to treat allergic asthma.

Elevated serum levels of TGF-β and increased production of TGF-β by unstimulated and PHA-stimulated PBMCs isolated from patients with asthma indicate that TGF-β may contribute to immunopathogenesis of allergic asthma. Therefore, targeting TGF-β or its related signaling pathways may have therapeutic benefits for allergic asthma.

The serum level of TGF-β in patients with severe asthma was higher than that of the ones with moderate asthma, whereas the TGF-β production by PHA-stimulated PBMCs isolated from patients with moderate asthma was also higher than that of the patients with severe asthma. The current study results showed that TGF-β may have different effects on moderate and severe asthma.

*GTAT3/FOXP3* expression ratio in patients with moderate asthma was significantly higher than that of the ones with severe asthma. Therefore, moderate and severe asthma may have significant differences in some main immunological parameters.
